# Inter- and intraspecific conflicts between parasites over host manipulation

**DOI:** 10.1098/rspb.2015.2870

**Published:** 2016-02-10

**Authors:** Nina Hafer, Manfred Milinski

**Affiliations:** Department of Evolutionary Ecology, Max Planck Institute for Evolutionary Biology, August-Thienemann-Straße 2, Plön, 24306 Germany

**Keywords:** host manipulation, interspecific conflict, intraspecific conflict, sabotage, cestode, nematode

## Abstract

Host manipulation is a common strategy by which parasites alter the behaviour of their host to enhance their own fitness. In nature, hosts are usually infected by multiple parasites. This can result in a conflict over host manipulation. Studies of such a conflict in experimentally infected hosts are rare. The cestode *Schistocephalus solidus* (S) and the nematode *Camallanus lacustris* (C) use copepods as their first intermediate host. They need to grow for some time inside this host before they are infective and ready to be trophically transmitted to their subsequent fish host. Accordingly, not yet infective parasites manipulate to suppress predation. Infective ones manipulate to enhance predation. We experimentally infected laboratory-bred copepods in a manner that resulted in copepods harbouring (i) an infective C plus a not yet infective C or S, or (ii) an infective S plus a not yet infective C. An infective C completely sabotaged host manipulation by any not yet infective parasite. An infective S partially reduced host manipulation by a not yet infective C. We hence show experimentally that a parasite can reduce or even sabotage host manipulation exerted by a parasite from a different species.

## 1. Introduction

Many parasites possess the ability to modify their host's behaviour or appearance to their needs. Such host manipulation has been reported from a large number of host–parasite systems (reviewed by [1–5]), including humans (e.g. [6,7]), and can have far reaching ecological consequences [8–12]. In complex life cycle parasites, many of the most prominent examples of host manipulation comprise cases in which parasites enhance the likelihood that their current host is consumed by a suitable subsequent host (reviewed by [1–5]). Normally, these changes do not occur before the parasite is ready for transmission. Premature predation would be fatal. Accordingly, parasites have developed the ability to lower their current host's predation risk prior to becoming infective to the next host [13–17]. Most studies of host manipulation, in particular under experimental conditions, focus on hosts infected with a single parasite species or even one individual. This does not reflect nature, where multiple infections are frequent (e.g. [18,19]). Most parasites do encounter several co-infecting parasites, potentially including other manipulating parasites. Host behaviour might influence even a non-manipulating parasite's fitness—its fitness might simply be highest in a normally behaving host and hampered by a co-infecting parasite's manipulation. This can result in a conflict over host manipulation, which is likely to alter how hosts are manipulated [14,20–26]. Nevertheless, only a few studies have explicitly investigated any conflict over host manipulation [27–32], and even fewer have used experimental infections [28,31,32].

Correlational evidence suggests that in co-infections in which there is potential for an interspecific conflict over host manipulation, both parasites can affect host behaviour [27,28,30]. Thomas *et al.* [28] found that hosts naturally infected with nematodes and trematodes are less manipulated than those exclusively infected by the trematodes. Cure and reinfection, however, failed to induce this effect [28]. Other studies using experimentally infected hosts have been restricted to an intraspecific conflict between infective and not yet infective parasite stages [31,32]. Hence, to our knowledge no evidence from experimentally infected hosts exists that one parasite can affect host manipulation by a heterospecific parasite. Such evidence, however, will be crucial to determine cause and effect if differences in behaviour are found between hosts harbouring different multi-species assemblages of parasites [26,33]. In a conflict between different developmental stages of the same species, studies on both naturally [29,31] and experimentally [31,32] infected hosts found that the infective parasite always had the stronger effect up to complete sabotage of the effect of the not yet infective parasite [32]. This raises the question whether, in such a conflict, the infective parasite might have a priority advantage and thus be generally able to interfere with the manipulation of a not yet infective, conspecific parasite. If so, would it be the case also in a conflict between two parasites from different species? They might use different mechanisms to manipulate making interference more difficult. However, parasites would benefit from altering their host in a manner that hinders any co-infecting parasite's manipulation that could reduce their fitness.

In this study, we use two phylogenetically distinct parasites that use cyclopoid copepods as their first intermediate hosts and fish as second intermediate hosts to investigate intra- and interspecific conflicts over host manipulation under strictly experimental conditions. The cestode *Schistocephalus solidus* has a three-host life cycle, with copepods as first and three-spined sticklebacks (*Gasterosteus aculeatus*) as second intermediate hosts, and piscivorous birds as definitive hosts [34,35]. Copepods infected by *S. solidus* have decreased activity [13,32,36] and predation susceptibility [16] until the parasite reaches infectivity. Once infective, *S. solidus* can increase host activity [13,37,38] and predation susceptibility [38]. The nematode *Camallanus lacustris* uses perch (*Perca fluviatilis*) as its definitive host. Other fish, including three-spined sticklebacks, can act as paratenic hosts [39]. Not yet infective *C. lacustris* reduce the predation susceptibility of their copepod host [16]. Behaviour that might be responsible for this change such as activity has not been measured directly in *C. lacustris* infected hosts. We expect a similar pattern of copepod activity as induced by *S. solidus* with an initial phase of decreased activity followed by increased activity. Accordingly, we do not expect an interspecific conflict over host manipulation between *S. solidus* and *C. lacustris* of the same developmental stage but between different stages when the two parasite species co-occur. Such a conflict does not require clear manipulation by both developmental stages; if normal host behaviour fits one parasite's need it should not manipulate itself but should interfere with counter-manipulation when the other parasite manipulates in the wrong direction. We focus on such a conflict between different developmental stages over host manipulation both within and between species. In this conflict, the infective parasite can sabotage manipulation by the not yet infective one.

## 2. Material and methods

### (a) Hosts

We used laboratory-bred copepods (*Macrocyclops albidus*) from a stock originating from the ‘Neustädter Binnenwasser’, northern Germany. We used adult male copepods to reduce variation between hosts. On the day prior to the first infection, 936 (experiment I) or 768 (experiment II) copepods were filtered from their tank and transferred each to an individual well of a 24-well cell culture plate in about 1 ml of water. Copepods were kept at 18°C in a 16 L : 8 D cycle. We checked for the presence of dead copepods, cleaned wells when necessary and fed the copepods with five *Artemia* sp. nautili every other day (i.e. the day when no behavioural recordings took place: days 1, 3, 5, 8, 10, 12, 14, 16, 18 and 20).

### (b) Parasites

*Camallanus lacustris* was dissected from perch guts obtained from a local fishery and originated from the Grosse Plöner See, northern Germany. To obtain gravid females, we cut open the blind sacks of perches' guts. Females were cleaned, placed in 0.64% sodium solution and stored in the fridge (4°C) until use. Gravid females harbour live larvae that are ready to infect copepods [39]. To obtain these larvae, we opened up the females with dissection needles, allowing the larvae to escape. Larvae were stored in tap water in the fridge overnight prior to copepod infections. A total of 40 (first infection, experiment I) and 30 (second infection, experiments I and II) females was used and their offspring mixed.

To obtain *S. solidus*, mature *S. solidus* were dissected from fish caught at the ‘Neustädter Binnenwasser’, northern Germany. They were bred *in vitro* in the laboratory [40] and eggs were stored in the fridge (4°C) until use. Prior to exposure the eggs were incubated for three weeks at 20°C and exposed to light over night to induce hatching [35]. *Schistocephalus solidus* stemmed from 2 (experiment I) or 4 (experiment II) families, which were equally distributed between all treatments.

### (c) Infections

**Figure 1.**
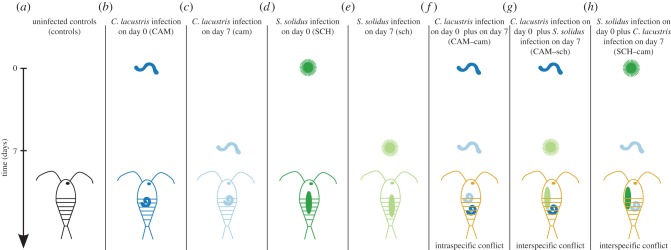
Overview over treatments and timing of infections. Copepods were exposed to *S. solidus*, *C. lacustris* or no parasite at two different time points, day 0 and day 7. This resulted in six different treatments for experiment I (control (*a*), CAM (*b*), cam (*c*), sch (*e*), CAM–sch (*f*), CAM–cam (*g*)) and four different treatments for experiment II (control (*a*), cam (*c*), SCH (*d*), SCH–cam (*h*)).

Infections consisted of adding either one coracidium (*S. solidus*) or one L1-larva (*C. lacustris*) to the well containing the copepod. This took place at two different time points, 7 days apart (i.e. on day 0 and on day 7). We conducted two experiments. In experiment I, we investigated either an intraspecific conflict between an infective *C. lacustris* and a not yet infective conspecific, or an interspecific conflict between an infective *C. lacustris* and a not yet infective *S. solidus*. Copepods were exposed to *C. lacustris* on day 0 and *S. solidus* or *C. lacustris* on day 7. Including the necessary controls, we obtained six different treatments (figure 1*a–c*, *e–g*): (*a*) not infected by any parasite (control), (*b*) infected by *C. lacustris* on day 0 (CAM), (*c*) infected by *C. lacustris* on day 7 (cam), (*e*) infected by *S. solidus* on day 7 (sch), (*f*) infected by *C. lacustris* on day 0 plus *C. lacustris* on day 7 (CAM–cam), (*g*) infected by *C. lacustris* on day 0 plus *S. solidus* on day 7 (CAM–sch). In experiment II, we investigated the potential conflict between an infective *S. solidus* and a not yet infective *C. lacustris*. Hence we used four different treatments (figure 1*a,c,d,h*): (*a*) not infected by any parasite (control), (*c*) infected by *C. lacustris* on day 7 (cam), (*d*) infected by *S. solidus* on day 0 (SCH) and (*h*) infected by *S. solidus* on day 0 plus *C. lacustris* on day 7 (SCH–cam). For each experiment, copepods from each treatment were spread evenly over all plates and distributed randomly.

At the end of the experiment, we checked whether an infection had occurred by placing copepods under a microscope. Because copepods are translucent, parasites within the copepod can be seen that way. We only checked for infection after all behavioural activities had been recorded to avoid stress that could have influenced copepod behaviour.

### (d) Recording of behaviour and analysis

Copepod behaviour was recorded by placing a plate with copepods on an apparatus that dropped it by about 3 mm [13,32]. This simulates a failed predator attack after which the copepod should perceive an enhanced predation risk since the predator might still be around. This simulated predator attack was initiated after the plate had been on the apparatus for 1 min. We used a Panasonic Super DynamicWV-BP550 camera (Panasonic Corporation, Osaka, Japan) to record copepod behaviour for 15 min after the simulated predator attack.

We analysed behaviour only of copepods that were infected by all parasites they had been exposed to. Copepods that died within 1 day after the last behavioural recordings (i.e. prior to day 22 in the experiment) were excluded from the analysis. If more than 40 copepods were available for one treatment, we randomly selected 40 subjects for analysis. This resulted in 240 copepods in experiment I (40 in each treatment) and 150 copepods in experiment II (C: 40; SCH: 30; cam: 40; SCH–cam: 40). Using the manual tracker plugin in ImageJ [41], we recorded the position of every copepod every 2 s for 1 min starting 10 s after the simulated predator attack to rule out the initial reaction and for 1 min at the end of the recording (between 14 and 15 min after the simulated predator attack). This was done blindly with regard to treatment. We assumed that copepods should have recovered from the simulated predator attack after 14 min. From the position data, we calculated whether or not a copepod had moved within each 2 s interval [32]. We also determined the latency for each copepod to resume moving after the simulated predator attack. If copepods did not move within 15 min, we assumed latency to be 15 min (14 out of 2716 behavioural recordings).

### (e) Statistical analysis

To investigate the effect of *C. lacustris* and *S. solidus* on host behaviour, we used generalized linear mixed models in the lme4 package [42] in R [43]. To account for variation between individual copepods over time, we included copepod identity and the day in the experiment (i.e. after the first infection on day 0) as random effects. To analyse copepod activity (i.e. whether or not a copepod moved within each two second interval), we additionally included the time point in the recording (i.e. after the simulated predator attack or after a recovery period) in the random effect. We fitted two separate models, one with activity as response variable using binomial distribution, the other one with the log-transformed latency to resume moving as response variable. We included the day and the time point as fixed effects. We stepwise added treatment and all its interactions with day and time point. We compared the models using AIC and used likelihood ratio tests to obtain *p*-values for this comparison. We accepted a model as having a better fit than a less complicated one if it explained the data significantly better. We fitted separate models for experiments I and II since they contained different treatments. Refer to the electronic supplementary material, tables S1 and S2 for the complete output of the models.

As we found significant interactions between treatment, day and time point (electronic supplementary material, tables S1 and S2) we conducted post hoc tests. We used Tukey tests with general linear hypotheses within the multcomp package in R to obtain *p*-values for each comparison that were adjusted for multiple testing [44]. We used separate post hoc tests for each treatment and time point to determine when significant changes occurred between consecutive days. Additionally, we used separate post hoc tests for each day and time point to investigate differences between treatments. In the Results section, we only report the most relevant statistics to facilitate readability. See the electronic supplementary material, tables S3–S6 for all other statistics.

## 3. Results

### (a) Host manipulation by *Camallanus lacustris*

**Figure 2.**
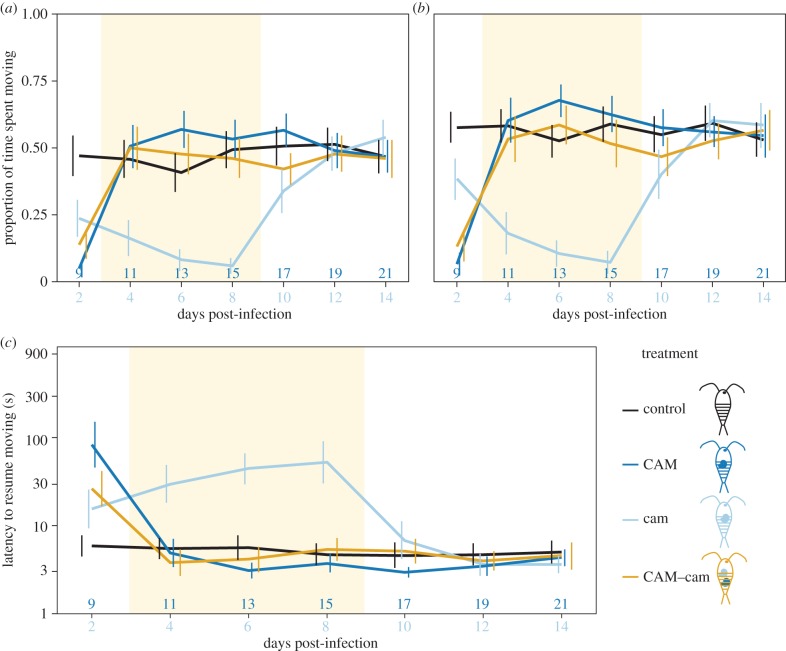
Intraspecific conflict within *Camallanus lacustris.* Error bars indicate 95% CI. (*a*) Activity (proportion of time spent moving) within 1 min after a simulated predator attack, (*b*) activity during 1 min after a recovery period, (*c*) latency to resume moving after a simulated predator attack. Note that the *y*-axis is on a log-scale. The upper labels on the *x*-axis indicate the age of *C. lacustris* from day 0, the lower labels indicate the age of *C. lacustris* from day 7. The coloured area indicates when a conflict over host manipulation should occur. *n* = 40 per treatment. Control, uninfected control copepods (figure 1*a*); CAM, copepods infected with *C. lacustris* on day 0 (figure 1*b*); cam, copepods infected with *C. lacustris* on day 7 (figure 1*c*); CAM–cam, copepods infected with one *C. lacustris* on day 0 plus one on day 7 (figure 1*f*).

We expect that prior to reaching infectivity (i.e. prior to 11 days post-infection, hereafter dpi) *C. lacustris* will suppress its host's predation risk (i.e. by reducing its activity and increasing its latency to resume moving after a simulated predator attack—stickleback predation on copepods correlates positively with copepod activity [38]) because too early predation would be fatal for the parasite [45]. Copepods that were infected by *C. lacustris* on day 7 (called ‘cam’, figure 1) were significantly less active than uninfected copepods between day 9 and 17 (i.e. 2–10 dpi; *p* < 0.02; electronic supplementary material, tables S5 and S6; figure 2*a,b*). This was preceded by a drop in host activity between consecutive days: between day 9 and 13 (i.e. 2–6 dpi; *p* < 0.001; electronic supplementary material, tables S3 and S4; figure 2*a,b*) in cam-copepods. In line with this, latency was significantly higher in cam-copepods than in uninfected ones. This was clearest from day 11 to day 15 (i.e. 4–8 dpi; *p* < 0.001; electronic supplementary material, table S5). Copepods that had been infected on day 0 (called ‘CAM’) were also less active and had a longer latency than uninfected control copepods on day 9 (i.e. 9 dpi, before their parasite reached infectivity; *p* < 0.001; electronic supplementary material, table S5; figure 2). Thus, we can confirm that copepods infected by not yet infective *C. lacustris* had a reduced activity and increased latency to resume moving compared with uninfected copepods, which is likely to result in reduced predation and may hence be termed predation suppression [45].

Once *C. lacustris* has reached infectivity to the next host (after 11 dpi), it should switch from predation suppression to predation enhancement [45], which should result in increased activity and decreased latency of copepods harbouring infective *C. lacustris* compared with uninfected copepods. In line with such a switch, cam-copepods' activity increased significantly between consecutive days as the parasite reached infectivity (day 15–19, i.e. 8–12 dpi; *p* < 0.001; electronic supplementary material, tables S3 and S4; figure 2*a,b*). In CAM-copepods activity increased around the same time post infection as in cam, between day 9 and 13 (i.e. 9 and 13 dpi; *p* < 0.02; electronic supplementary material, tables S3 and S4; figure 2*a,b*). As the parasite became infective (i.e. between 8 and 11 dpi), latency decreased in both cam-copepods (day 15–17; electronic supplementary material, tables S3 and S4; figure 2*c*) and CAM–copepods (day 9–11, *p* < 0.001; electronic supplementary material, table S3; figure 2*c*). These changes mostly resulted in slightly higher activity and shorter latency of CAM-copepods than those of uninfected copepods (figure 2), although these differences were only significant on some days; specifically, activity on day 13 in CAM-copepods (*p* < 0.04; electronic supplementary material, table S5) and activity after a simulated predator attack (*p* = 0.039) and latency (*p* < 0.001) on day 19 (i.e. 12 dpi) in cam-copepods in experiment II (electronic supplementary material, table S6). In conclusion, we found increased activity potentially indicative of predation enhancement [45], albeit it was much less pronounced than potential predation suppression.

### (b) Intraspecific conflict between two *Camallanus lacustris* parasites

If two parasites manipulate differently or one manipulates and for the other normal host behaviour would be optimal, there is potential for a conflict over host manipulation between them. To investigate this potential conflict between different developmental stages of *C. lacustris*, we used copepods infected with *C. lacustris* on day 0 (CAM, figure 1*b*) or day 7 (cam, figure 1*c*) and copepods infected with *C. lacustris* on day 0 plus on day 7 (called ‘CAM–cam’, figure 1*f*). To establish when such a conflict would occur we compared CAM-copepods with cam-copepods. From day 11 to day 17 (i.e. when CAM was already infective but cam was not yet), CAM-copepods were significantly more active and resumed moving sooner than cam-copepods (*p* < 0.007; electronic supplementary material, table S5; figure 2), mostly because cam-copepods strongly manipulated to lower host activity (see above). Hence, during this time we could expect a conflict between two such parasites if they infected the same host. CAM–cam-copepods were significantly more active and resumed moving sooner than cam-copepods from day 11 to day 15 (*p* < 0.001; electronic supplementary material, table S5; figure 2). During this time, the behaviour of CAM-copepods did not differ significantly from that of CAM–cam-copepods (*p* > 0.4; electronic supplementary material, table S5; figure 2). On day 17, we observed no further differences between cam and CAM–cam-copepods (*p* > 0.2; electronic supplementary material, table S5; figure 2) nor between CAM-copepods and CAM–cam-copepods with regard to latency and activity after a recovery period (*p* > 0.3; electronic supplementary material, table S5), albeit CAM-copepods tended to be more active right after the simulated predator attack (*p* = 0.060; electronic supplementary material, table S5). Thus, the not yet infective parasite (cam) had no observable effect when together with an infective parasite (CAM). In accordance with this finding, CAM–cam-copepods increased their activity and decreased their latency between days 9 and 11 (*p* < 0.001; electronic supplementary material, table S3; figure 2)—as CAM reached infectivity. No further significant increases occurred after day 15, when cam should have reached infectivity and hence caused a switch in host behaviour (see above). We hence found that this intraspecific conflict was clearly won by the infective parasite.

### (c) Interspecific conflict between an infective *Camallanus lacustris* parasite and a not yet infective *Schistocephalus solidus* parasite

**Figure 3.**
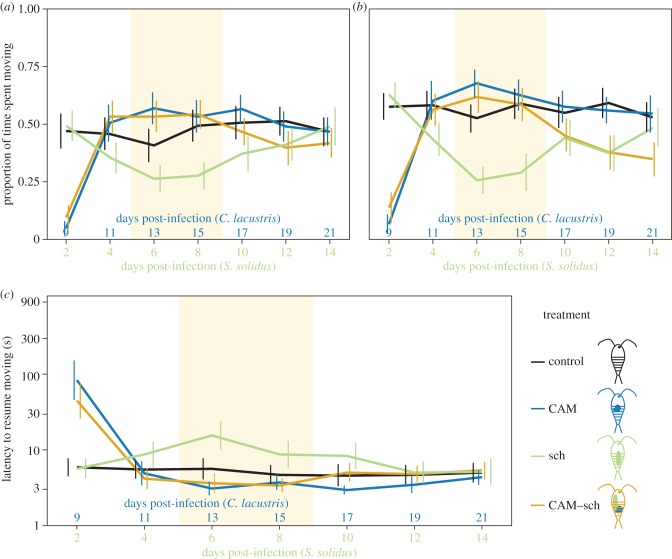
Interspecific conflict between an infective *Camallanus lacustris* and a not yet infective *Schistocephalus solidus*. Error bars indicate 95% CI. (*a*) Activity (proportion of time spent moving) within 1 min after a simulated predator attack, (*b*) activity during one minute after a recovery period, (*c*) latency to resume moving after a simulated predator attack. Note that the *y*-axis is on a log-scale. The coloured area indicates when a conflict over host manipulation should occur. *n* = 40 per treatment. Control, uninfected control copepods (figure 1*a*); CAM, copepods infected with *C. lacustris* on day 0 (figure 1*b*); sch, copepods infected with *S. solidus* on day 7 (figure 1*e*); CAM–sch, copepods infected with one *C. lacustris* on day 0 plus one *S. solidus* on day 7 (figure 1*f*).

To investigate a potential interspecific conflict between an infective *C. lacustris* and a not yet infective *S. solidus*, we used copepods infected with *C. lacustris* on day 0 (CAM, figure 1*b*), copepods infected with *S. solidus* on day 7 (sch, figure 1*e*) and copepods infected with *C. lacustris* on day 0 plus *S. solidus* on day 7 (called ‘CAM–sch’, figure 1*g*). Again, in order to establish the time window during which a conflict over host manipulation may occur, we first compared the behaviour of CAM-copepods to sch-copepods. During days 13 and 15, CAM-copepods were significantly more active and resumed moving sooner than sch-copepods (*p* < 0.02; electronic supplementary material, table S5; figure 3). These differences were driven by host manipulation prior to reaching infectivity in sch-copepods (i.e. reduced activity on days 13 and 15; *p* < 0.02; electronic supplementary material, table S5; figure 3*a,b*) and increased latency on day 13 (*p* < 0.001; electronic supplementary material, table S5; figure 3*c*), while CAM alone showed only very weak host manipulation (see above). During this time, CAM was infective and sch was not, so a conflict should have existed between them if they infected the same host. CAM–sch-copepods behaved significantly different from sch-copepods on days 13 and 15 (*p* < 0.006; electronic supplementary material, table S5) but seemed undistinguishable from CAM-copepods during this time (*p* > 0.8; electronic supplementary material, table S5; figure 3). Also alike CAM-copepods, CAM–sch-copepods increased their activity and decreased their latency as CAM reached infectivity between day 9 and day 11 (*p* < 0.001; electronic supplementary material, table S3; figure 3). Again, the infective parasite, CAM, seemed to win the conflict over host manipulation.

### (d) Interspecific conflict between an infective *Schistocephalus solidus* parasite and a not yet infective *Camallanus lacustris* parasite

**Figure 4.**
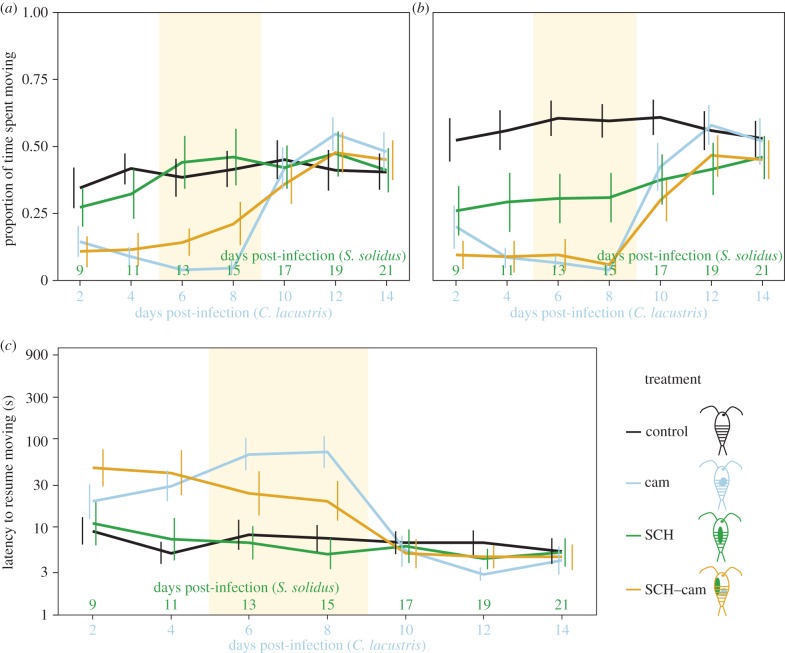
Interspecific conflict between an infective *Schistocephalus solidus* and a not yet infective *Camallanus lacustris*. Error bars indicate 95% CI. (*a*) Activity (proportion of time spent moving) within 1 min after a simulated predator attack, (*b*) activity during 1 min after a recovery period, (*c*) latency to resume moving after a simulated predator attack. Note that the *y*-axis is on a log-scale. The coloured area indicates when a conflict over host manipulation should occur. *n* = 40 for control, cam, SCH–cam; *n* = 30 for SCH. Control, uninfected control copepods (figure 1*a*); cam, copepods infected with *C. lacustris* on day 7 (figure 1*c*); SCH, copepods infected with *S. solidus* on day 0 (figure 1*d*); SCH–cam, copepods infected with one *S. solidus* on day 0 plus one *C. lacustris* on day 7 (figure 1*h*).

To study a conflict over host manipulation between an infective *S. solidus* and a not yet infective *C. lacustris*, we used copepods infected with *S. solidus* on day 0 (SCH, figure 1*d*), copepods infected with *C. lacustris* on day 7 (cam, figure 1*c*) and copepods infected with *S. solidus* on day 0 plus *C. lacustris* on day 7 (called ‘SCH–cam’, figure 1*h*). We tested for the existence and timing of this conflict by comparing SCH-copepods to cam-copepods. SCH-copepods were significantly more active than cam-copepods and resumed moving sooner between day 11 and 15 (i.e. 11 and 15 dpi for *S. solidus*, and 4 and 8 dpi for *C. lacustris*; *p* < 0.001; electronic supplementary material, table S6; figure 4). Again, the not yet infective parasite, in this case cam, drove these differences through its manipulation (see above) while hosts with infective *S. solidus* behaved similarly to controls after the simulated predator attack (*p* > 0.7; electronic supplementary material, table S6) and were less active than uninfected copepods after a recovery period (*p* < 0.001; electronic supplementary material, table S6). Hence a conflict seemed to exist between day 11 and day 15. However, day 11 should be considered with caution since a significant increase in host activity in SCH occurred only between days 11 and 13 (electronic supplementary material, table S4). This increase is likely to coincide with when *S. solidus* became infective and hence should increase its host's predation susceptibility (i.e. from day 13 onwards).

The behaviour of SCH–cam-copepods in which a conflict over host manipulation occurred was somewhat intermediate between that of SCH- and cam-copepods on days 13 and 15 (i.e. during the time when we expected a conflict). SCH–cam-copepods were more active than cam-copepods*,* after a simulated predator attack (*p* < 0.001; electronic supplementary material, table S6; figure 4*a*), but not after a recovery period (*p* > 0.8; electronic supplementary material, table S6), but always less active than SCH-copepods (*p* < 0.001; figure 4*a,b*; electronic supplementary material, table S6). Likewise, SCH–cam-copepods had a shorter latency than cam-copepods (*p* < 0.02; figure 4*c*; electronic supplementary material, table S6), but a longer latency than SCH-copepods (*p* < 0.002; figure 4*c*; electronic supplementary material, table S6). A conflict between SCH and cam seemed to result in an intermediate phenotype when it came to host manipulation.

## 4. Discussion

The nematode *Camallanus lacustris* initially alters host behaviour to arguably reduce predation on its host before it reaches infectivity. Thereafter slight changes of host behaviour into the opposite direction occur. This follows a pattern previously predicted [45] and shown in other systems [13,15,32,46]. The manipulation by *C. lacustris* is similar to but more pronounced than that of the cestode *Schistocephalus solidus* in the same copepod host [13,32]. Nevertheless, host manipulation by *C. lacustris* and *S. solidus* result in a similar reduction of predation susceptibility [16]. Different complex life cycle parasites that exploit the same trophic link also adopt convergent life-history strategies [47], suggesting that their host manipulation should also cause similar host behaviour.

Once we have shown that there is conflict (be it between two manipulating or one manipulating and one not manipulating parasite), we focus on the outcome of this conflict. This is where our main findings are. In any study to date that investigated an intraspecific conflict between different parasite stages, the infective parasite seems to win [29,31,32]. This seems to be the case even when the not yet infective parasite manipulates strongly and the infective one shows little clear host manipulation when each is alone [32]. We cannot rule out that *C. lacustris* or *S. solidus* also affect host behaviours other than activity and latency to recover after a simulated predator attack, with a modulating effect on what we measure. However, activity of copepods is a predictor of predation susceptibility to sticklebacks [38]. Hence we expect that the changes in activity we observed should result in changes in predation susceptibility. At least with regard to changes in host activity and latency, it seems that being the first to infect a host allows the infective parasite to become superior. This presents a puzzle. If the not yet infective parasite does manipulate when alone, it cannot benefit from ceasing to do so in the presence of an infective conspecific [32]. In *C. lacustris*, a not yet infective parasite is faced with the same problem. Any premature predation would be fatal. Accordingly, a not yet infective *C. lacustris* strongly reduces host activity when alone. Yet if it shares its host with an infective conspecific, it has no detectable manipulation effect.

In an interspecific conflict between *C. lacustris* and *S. solidus*, *C. lacustris* always does better. It is also the stronger manipulator. If there is a conflict over host manipulation between an infective *C. lacustris* and a not yet infective *S. solidus*, the infective *C. lacustris*, just as in an intraspecific conflict, seems to completely sabotage any host manipulation by *S. solidus*. A conflict between an infective *S. solidus* and a not yet infective *C. lacustris* results, however, in an intermediate phenotype. Overall, in both cases the infective parasite performs better in interspecific conflicts. *Camallanus lacustris* completely dominates host behaviour when infective but manages to settle for an intermediate phenotype when not yet infective. An infective *S. solidus* only partly increases host activity when sharing with a not yet infective *C. lacustris*, while the not yet infective *S. solidus* seems to have no effect at all.

The dominance of the infective parasite is particularly interesting when we consider the fact that, in contrast to the not yet infective parasite, it shows weak manipulation at most when alone. If the host's normal behaviour fits the infective parasite's needs, it should interfere when a non-infective parasite manipulates in the wrong direction, which it does. Is an infective parasite's ability to manipulate host behaviour inherently stronger than a not yet infective one's? Infective parasites are bigger. Thus, if for instance they manipulate by secreting some substance, they would probably be able to produce larger quantities. We did not test the effect of parasite number. However, in an intraspecific conflict within *S. solidus*, multiple not yet infective *S. solidus* were as unable as a single one to prevent the sabotage of their manipulation by one infective conspecific [32]. It seems unlikely that the better performance of the infective parasite is due to its size. At least in parasites which switch their host manipulation from predation suppression to predation enhancement such as *S. solidus* and *C. lacustris*, the infective parasite could benefit from switching off its own initial predation suppression. Such a switch could also affect a not yet infective conspecific's manipulation [32]. Very little is known about the mechanisms underlying host manipulation by either *S. solidus* or *C. lacustris* in copepods. Host manipulation can occur through physiological side-effects, encystment at a certain site, hitchhiking the immune system or neuromodulation (e.g. [48–53]). Encystment at a certain site can be ruled out because both species occur at various positions in the body cavity. Side effects through enhanced energy drain have been proposed as a mechanism frequently used by cestodes to manipulate their hosts [50], including for *S. solidus* in its stickleback host (e.g. [54–56]). However, an infection with *S. solidus* does not seem to cause any resource depletion [57] and its manipulation does not seem directly affected by resource availability [58] in copepods. Other physiological constraints would seem possible [53], but it seems doubtful whether such constraints could just cease once a parasite has reached infectivity or its host is co-infected by an infective parasite. They would have to be counteracted by some other mechanism.

In interspecific interactions, the result looks similar to intraspecific interaction; the infective parasite sabotages the manipulation of the non-infective other parasite completely when *C. lacustris* is the infective parasite; however, it does so only partly when *S. solidus* is the infective parasite. Here is a difference between the two parasite species, which is consistent with our finding that *C. lacustris* is the stronger manipulator. We neither need to postulate that an infective parasite has evolved a specific mechanism to dominate a not yet infective conspecific, it actively stops its own manipulation, and as a side effect that of the conspecific, nor do we need to postulate an evolved mechanism for dominating a not yet infective heterospecific. If both *S. solidus* and *C. lacustris* use a very similar mode of active manipulation to decrease host activity, it could easily be sabotaged also interspecifically. It has previously been shown that even distantly related parasites seem sometimes to use the same proximate mechanism to manipulate their hosts [59]. This seems to be the most parsimonious explanation of our complete dataset. Accordingly, for this explanation it is not important that both intraspecific and interspecific competition between an infective and a not yet infective parasite had occurred often enough so that specific mechanisms could evolve. The necessary mechanism had already been evolved for switching from decreasing to increasing the host's activity by the same individual parasite in the optimal time window [45]. While the effectiveness of this switch against co-infecting parasites could have originated as a side effect, it nevertheless presents an evolutionary advantage for the infective parasite preventing unsuitable manipulation of the host by other co-infecting parasites.

Once a host is infected by a parasite, this infected and potentially manipulated host will present an environment different from a healthy host [2,10,60]. Here we show for the first time using experimental infections that a parasite can influence and even completely sabotage host manipulation by a parasite from a different species. Host manipulation can have important ecological consequences [8–12], for example by altering trophic links in a food web [10,12]. In addition, it can have severe medical implications (e.g. *Toxoplasma gondii* [6,7] or malaria [61,62]). Given the abundance of (manipulating) parasites, interactions among a multitude per host are likely to determine host behaviour in nature. The host might be a ‘puppet on the string’ moved by its many different parasites.

## Supplementary Material

SI tables

## Data accessibility

All data have been submitted to the dryad repository: http://dx.doi.org/10.5061/dryad.4061p.
