# Supplementary material for: Inter- and intraspecific conflicts between parasites over host manipulation
Source: Proc Biol Sci. 2016 Feb 10;283(1824):20152870. doi: 10.1098/rspb.2015.2870 (PMC4760176; doi:10.1098/rspb.2015.2870)
Supplement: SI tables [file rspb20152870supp1.pdf]

Supplementary information

Table S1: Outcome of likelihood ratio tests for copepod activity (i.e., proportion of time spent moving within one minute). The initial model used whether or not a copepod moved within a two second interval as response and the day after the first infection (DAY), the Period in the recording (PERIOD), i.e. after a simulated predator attack vs. after a recovery period and the interaction between DAY and TIME as fixed effects. We used the copepod identity as a random factor and included DAY and PERIOD (AIC: Experiment I: 126326, Experiment II: 66973). Subsequently, we added the treatment (TREAT) and all its interactions with DAY and PERIOD. Test statistics and MCMC-estimated p-values are for the comparison with the preceding model.

Experiment I				
Factors	AIC	DF	Chisq	p
+ TREAT	126248	15,5	88.522	<0.0001
+ DAY:TREAT	126177	20,5	80.475	<0.0001
+ PERIOD:TREAT	126160	25,5	27.401	<0.0001
+ PERIOD:DAY:TREAT	126117	30,5	53.011	<0.0001
100800 observations on 240 copepods				

Experiment II				
Factors	AIC	DF	Chisq	p
+ TREAT	66941	13,3	38.223	<0.0001
+ DAY:TREAT	66872	16,3	74.504	<0.0001
+ PERIOD:TREAT	66770	19,3	108.823	<0.0001
+ PERIOD:DAY:TREAT	66747	22,3	28.672	<0.0001
62160 observations on 150 copepods				

Table S2: Outcome of likelihood ratio tests for latency to resume moving after a simulated predation attack. The initial model used the time when a copepod first moved following a 10 second interval after the simulated predator attack and the day after the first infection (DAY) as fixed effect. We used the copepod identity as a random factor and included DAY (AIC: Experiment I: 5191, Experiment II: 3309). Subsequently, we added the treatment (TREAT) and its interactions with DAY. Test statistics and MCMC-estimated p-values are for the comparison with the preceding model.

Experiment I						Experiment II				
Factors	AIC	DF	Chisq	p		Factors	AIC	DF	Chisq	p
+ TREAT	5116	11,5	84.623	<0.0001		+ TREAT	3281	9,3	33.871	<0.0001
+ DAY:TREAT	5066	16,5	60.699	<0.0001		+ DAY:TREAT	3229	12,3	58.168	<0.0001
1680 observations on 240 copepods						1036 observations on 150 copepods				

Table S3: Outcome of multiple comparisons between days for each treatment. Results from experiment I. Significant p-values are highlighted in bold. Control: Uninfected control copepods, CAM: Copepods infected with *C. lacustris* on day 0, cam: Copepods infected with *C. lacustris* on day 7, sch: Copepods infected with *S. solidus* on day 7, CAM-cam: Copepods infected with one *L. lacustris* on day 0 plus one on day 7, CAM-sch: Copepods infected with one *L. lacustris* on day 0 plus one *S. solidus* on day 7.

**Activity after simulated predator attack**												
Treatment	Control		CAM		cam		sch		CAM-cam		CAM-sch	
Comparison	Z	p	Z	p	Z	p	Z	p	Z	p	Z	p
day9-day11	0.34	1	24.50	**<0.001**	-11.34	**<0.001**	-9.63	**<0.001**	20.05	**<0.001**	21.03	**<0.001**
day11-day13	-2.98	**0.045**	3.94	**0.002**	-5.43	**<0.001**	-9.35	**<0.001**	2.61	0.122	2.91	0.055
day13-day15	3.29	**0.018**	-2.76	0.080	-2.85	0.064	1.88	0.497	-3.48	**0.009**	-1.77	0.569
day15-day17	-1.97	0.434	-2.61	0.118	17.80	**<0.001**	7.89	**<0.001**	-2.50	0.155	-6.70	**<0.001**
day17-day19	2.14	0.327	-0.82	0.982	10.35	**<0.001**	-3.38	**0.013**	2.92	0.053	-3.81	**0.003**
day19-day21	-3.16	**0.026**	-0.77	0.987	-0.96	0.961	5.55	**<0.001**	2.05	0.378	-1.54	0.721
Observations	8400		8400		8400		8400		8400		8400	
Copepods	40		40		40		40		40		40	

**Activity after a recovery period**												
Treatment	Control		CAM		cam		sch		CAM-cam		CAM-sch	
Comparison	Z	p	Z	p	Z	p	Z	p	Z	p	Z	p
day9-day11	-0.64	0.996	21.26	**<0.001**	-4.67	**<0.001**	-7.06	**<0.001**	18.22	**<0.001**	21.34	**<0.001**
day11-day13	-2.61	0.122	3.24	**0.019**	-6.04	**<0.001**	-4.95	**<0.001**	-1.12	0.920	0.04	1
day13-day15	4.39	**<0.001**	-1.75	0.568	-2.08	0.350	0.76	0.989	-0.75	0.989	0.38	1
day15-day17	0.68	0.994	1.58	0.683	15.69	**<0.001**	5.02	**<0.001**	-2.05	0.378	-3.81	**0.003**
day17-day19	0.34	1	-3.83	**0.002**	7.20	**<0.001**	2.16	0.317	2.89	0.059	-3.51	**0.008**
day19-day21	-2.30	0.246	-1.11	0.922	2.99	**0.042**	3.98	**0.001**	-0.96	0.962	1.11	0.924
Observations	8400		8400		8400		8400		8400		8400	
Copepods	40		40		40		40		40		40	

Latency to resume moving after a simulated predator attack												
Treatment	Control		CAM		cam		sch		CAM-cam		CAM-sch	
Comparison	Z	p	Z	p	Z	p	Z	p	Z	p	Z	p
day9-day11	-0.28	1	-12.68	**<0.001**	2.08	0.366	1.72	0.604	-8.07	**<0.001**	-10.32	**<0.001**
day11-day13	0.18	1	-1.83	0.526	1.21	0.891	2.26	0.261	0.30	1	-0.48	0.999
day13-day15	-0.81	0.984	0.71	0.992	0.74	0.990	-2.20	0.293	0.78	0.987	-0.42	1
day15-day17	0.01	1	-0.92	0.969	-6.41	**<0.001**	-0.18	1	-0.10	1	1.52	0.731
day17-day19	-0.02	1	0.73	0.991	-2.04	0.388	-1.85	0.516	-1.02	0.949	-0.24	1
day19-day21	0.26	1	0.67	0.994	-0.02	1	0.20	1	0.62	0.996	0.39	1
Observations	280		280		280		280		280		280	
Copepods	40		40		40		40		40		40	

Table S4: Outcome of multiple comparisons between days for each treatment. Results from experiment II. Significant p-values are highlighted in bold. Control: Uninfected control copepods, SCH: Copepods infected with *S. solidus* on day 0, cam: Copepods infected with *C. lacustris* on day 7, SCH-cam: Copepods infected with one *S. solidus* on day 0 plus one *C. lacustris* on day 7.

Activity after simulated predator attack

Treatment	Control		SCH		cam		SCH-cam	
Comparison	Z	p	Z	p	Z	p	Z	p
day9-day11	3.78	**0.003**	2.24	0.274	-4.17	**0.001**	0.98	0.957
day11-day13	-1.78	0.560	5.47	**<0.001**	-4.79	**<0.001**	1.58	0.687
day13-day15	1.68	0.629	0.89	0.974	0.80	0.984	4.55	**<0.001**
day15-day17	1.79	0.554	-1.49	0.751	18.48	**<0.001**	8.62	**<0.001**
day17-day19	-2.09	0.358	1.98	0.429	6.42	**<0.001**	5.33	**<0.001**
day19-day21	-0.13	1	-2.72	0.094	-3.33	**0.014**	-1.17	0.903
Observations	8340		6240		8250		8250	
Copepods	40		30		40		40	

Activity after a recovery period

Treatment	Control		SCH		cam		SCH-cam	
Comparison	Z	p	Z	p	Z	p	Z	p
day9-day11	1.97	0.434	1.54	0.721	-7.86	**<0.001**	-0.29	1
day11-day13	2.20	0.294	0.68	0.994	-1.67	0.617	0.22	1
day13-day15	-0.48	0.999	0.16	1	-2.81	0.068	-3.20	**0.022**
day15-day17	0.65	0.995	3.39	**0.012**	18.73	**<0.001**	14.67	**<0.001**
day17-day19	-2.58	0.131	1.53	0.728	8.13	**<0.001**	8.17	**<0.001**
day19-day21	-1.38	0.811	1.94	0.455	-3.17	**0.023**	-0.61	0.996
Observations	8340		6240		8250		8250	
Copepods	40		30		40		40	

Latency to resume moving after a simulated predator attack

Treatment	Control		SCH		cam		SCH-cam	
Comparison	Z	p	Z	p	Z	p	Z	p
day9-day11	-2.54	0.145	-1.24	0.880	1.36	0.823	-0.33	1
day11-day13	2.18	0.304	-0.46	0.999	3.17	**0.025**	-1.68	0.631
day13-day15	-0.47	0.999	-0.86	0.978	0.27	1	-0.68	0.994
day15-day17	-0.62	0.996	0.54	0.998	-9.15	**<0.001**	-4.16	**0.001**
day17-day19	0.07	1	-1.11	0.926	-2.02	0.404	-0.27	1
day19-day21	-0.82	0.983	0.65	0.995	1.16	0.909	0.04	1
Observations	278		275		208		275	
Copepods	40		40		30		40	

Table S5: Outcome of multiple comparisons between treatments for each day. Results from experiment I. Significant p-values are highlighted in bold. Control: Uninfected control copepods. CAM: Copepods infected with *C. lacustris* on day 0. cam: Copepods infected with *C. lacustris* on day 7. sch: Copepods infected with *S. solidus* on day 7. CAM-cam: Copepods infected with one *L. lacustris* on day 0 plus one on day 7. CAM-sch: Copepods infected with one *L. lacustris* on day 0 plus one *S. solidus* on day 7.

**Activity after simulated predator attack**														
Day	9		11		13		15		17		19		21	
	Z	p	Z	p	Z	p	Z	p	Z	p	Z	p	Z	p
Control _CAM	-9.41	**<0.001**	0.79	0.969	3.03	**0.030**	0.73	0.978	1.22	0.828	-0.35	0.999	0.10	1
Control _cam	-4.30	**<0.001**	-6.71	**<0.001**	-8.64	**<0.001**	-11.40	**<0.001**	-3.64	**0.004**	-0.65	0.987	1.38	0.738
Control _sch	0.34	0.999	-1.84	0.442	-3.16	**0.019**	-4.50	**<0.001**	-2.66	0.084	-2.06	0.307	0.28	1
Control _CAM-cam	-6.52	**<0.001**	0.69	0.983	1.19	0.844	-0.69	0.983	-1.56	0.623	-0.64	0.988	-0.18	1
Control _CAM-sch	-7.95	**<0.001**	1.23	0.820	2.21	0.234	0.91	0.944	-0.73	0.978	-2.35	0.174	-0.89	0.949
CAM_cam	-5.60	**<0.001**	7.44	**<0.001**	11.37	**<0.001**	12.02	**<0.001**	4.85	**<0.001**	0.30	1	-1.28	0.795
CAM_sch	9.70	**<0.001**	-2.62	0.093	-6.14	**<0.001**	-5.22	**<0.001**	-3.87	**0.002**	-1.71	0.525	0.18	1
CAM_CAM-cam	3.43	**0.008**	-0.10	1	-1.84	0.437	-1.42	0.716	-2.79	0.060	-0.29	1	-0.28	1
CAM_CAM-sch	1.79	0.469	0.44	0.998	-0.83	0.962	0.18	1	-1.95	0.370	-2.00	0.344	-0.99	0.921
cam_sch	4.64	**<0.001**	4.95	**<0.001**	5.67	**<0.001**	7.43	**<0.001**	1.00	0.918	-1.42	0.717	-1.10	0.883
cam_CAM_cam	-2.32	0.186	7.35	**<0.001**	9.72	**<0.001**	10.80	**<0.001**	2.11	0.283	0.01	1	-1.56	0.627
cam_CAM-sch	-3.93	**0.001**	7.87	**<0.001**	10.65	**<0.001**	12.19	**<0.001**	2.94	**0.039**	-1.70	0.530	-2.27	0.207
sch_CAM-cam	6.85	**<0.001**	-2.52	0.118	-4.33	**<0.001**	-3.81	**0.002**	-1.11	0.878	-1.42	0.713	0.45	0.998
sch_CAM_sch	8.27	**<0.001**	-3.06	**0.027**	-5.34	**<0.001**	-5.40	**<0.001**	-1.94	0.376	0.29	1	1.16	0.855
CAM-cam_CAM_sch	-1.67	0.551	0.54	0.995	1.02	0.911	1.60	0.598	0.84	0.961	-1.71	0.526	-0.71	0.981
Observations	7200		7200		7200		7200		7200		7200		7200	
Copepods	240		240		240		240		240		240		240	

**Activity after a recovery period**														
Day	9		11		13		15		17		19		21	
Control _CAM	-10.57	**<0.001**	0.22	1	2.98	**0.034**	1.03	0.875	0.49	0.997	-0.60	0.991	0.31	1
Control _cam	-3.17	**0.019**	-8.26	**<0.001**	-9.88	**<0.001**	-6544.67	**<0.001**	-3.04	**0.029**	0.09	1	0.93	0.939
Control _sch	0.79	0.969	-2.69	0.076	-5.31	**<0.001**	-7.72	**<0.001**	-1.98	0.354	-4.41	**<0.001**	-0.94	0.936
Control _CAM-cam	-8.63	**<0.001**	-0.78	0.971	1.00	0.918	-1.82	0.380	-1.36	0.751	-1.21	0.830	0.57	0.993
Control _CAM-sch	-8.80	**<0.001**	-0.47	0.997	1.73	0.511	-0.01	1	-1.68	0.544	-4.23	**<0.001**	-3.13	**0.022**
CAM_cam	-7.79	**<0.001**	8.44	**<0.001**	12.46	**<0.001**	18.86	**<0.001**	3.51	**0.006**	-0.69	0.983	-0.61	0.990
CAM_sch	11.24	**<0.001**	-2.90	**0.043**	-8.18	**<0.001**	-6.26	**<0.001**	-2.46	0.135	-3.82	**0.002**	-1.25	0.813
CAM_CAM-cam	2.40	0.156	-1.00	0.920	-1.98	0.352	-2.02	0.269	-1.85	0.435	-0.62	0.990	0.26	1
CAM_CAM-sch	2.07	0.302	-0.69	0.983	-1.26	0.808	-0.74	0.967	-2.17	0.254	-3.64	**0.004**	-3.43	**0.008**
cam_sch	3.95	**0.001**	5.70	**<0.001**	4.93	**<0.001**	9.52	**<0.001**	1.07	0.894	-4.50	**<0.001**	-1.86	0.427
cam_CAM_cam	-5.65	**<0.001**	7.50	**<0.001**	10.76	**<0.001**	15.95	**<0.001**	1.69	0.538	-1.30	0.783	-0.35	0.999
cam_CAM-sch	-5.89	**<0.001**	7.82	**<0.001**	11.39	**<0.001**	17.86	**<0.001**	1.37	0.745	-4.31	**<0.001**	-4.04	**0.001**
sch_CAM-cam	9.35	**<0.001**	-1.91	0.396	-6.28	**<0.001**	-4.27	**<0.001**	-0.62	0.989	-3.21	**0.017**	-1.51	0.659
sch_CAM_sch	9.50	**<0.001**	-2.23	0.225	-6.98	**<0.001**	-5.54	**<0.001**	-0.30	1	-0.19	1	2.18	0.248
CAM-cam_CAM_sch	-0.32	1	0.31	1	0.73	0.978	1.28	0.739	-0.32	1	-3.02	**0.030**	-3.69	**0.003**
Observations	7200		7200		7200		7200		7200		7200		7200	
Copepods	240		240		240		240		240		240		240	

Latency to resume moving after a simulated predator attack														
Day	9		11		13		15		17		19		21	
Control _CAM	8.05	**<0.001**	-0.25	1	-2.13	0.270	-0.82	0.964	-1.76	0.491	-1.38	0.740	-0.72	0.980
Control _cam	2.91	**0.042**	6.59	**<0.001**	8.53	**<0.001**	9.85	**<0.001**	1.78	0.477	-1.23	0.823	-1.38	0.739
Control _sch	-0.09	1	1.74	0.502	4.10	**0.001**	2.36	0.170	2.28	0.203	0.43	0.998	0.40	0.999
Control _CAM-cam	4.41	**<0.001**	-1.04	0.906	-0.97	0.928	0.38	0.999	0.30	1	-0.90	0.948	-0.34	0.999
Control _CAM-sch	6.13	**<0.001**	-0.80	0.967	-1.46	0.690	-1.20	0.835	0.21	1	-0.02	1	0.19	1
CAM_cam	5.14	**<0.001**	-6.84	**<0.001**	-10.66	**<0.001**	-10.67	**<0.001**	-3.54	**0.005**	-0.15	1	0.66	0.986
CAM_sch	-8.14	**<0.001**	1.99	0.348	6.23	**<0.001**	3.18	**0.018**	4.04	**0.001**	1.81	0.462	1.12	0.874
CAM_CAM-cam	-3.64	**0.004**	-0.79	0.969	1.16	0.854	1.20	0.836	2.06	0.309	0.48	0.997	0.38	0.999
CAM_CAM-sch	-1.92	0.391	-0.56	0.994	0.67	0.985	-0.38	0.999	1.97	0.358	1.36	0.750	0.91	0.945
cam_sch	-3.00	**0.032**	-4.85	**<0.001**	-4.43	**<0.001**	-7.49	**<0.001**	0.50	0.996	1.66	0.561	1.78	0.477
cam_CAM_cam	1.50	0.666	-7.63	**<0.001**	-9.50	**<0.001**	-9.47	**<0.001**	-1.48	0.674	0.33	0.999	1.04	0.903
cam_CAM-sch	3.22	**0.016**	-7.40	**<0.001**	-9.99	**<0.001**	-11.06	**<0.001**	-1.57	0.618	1.21	0.832	1.57	0.616
sch_CAM-cam	-4.50	**<0.001**	2.78	0.061	5.06	**<0.001**	1.98	0.355	1.98	0.354	1.32	0.772	0.74	0.977
sch_CAM_sch	-6.22	**<0.001**	2.55	0.111	5.56	**<0.001**	3.57	**0.005**	2.07	0.305	0.44	0.998	0.21	1
CAM-cam_CAM_sch	1.72	0.518	0.23	1	-0.49	0.996	-1.59	0.607	-0.09	1	0.88	0.952	0.53	0.995
Copepods	240		240		240		240		240		240		240	

Table S6: Outcome of multiple comparisons between treatments for each day. Results from experiment II. Significant p-values are highlighted in bold. Control: Uninfected control copepods, SCH: Copepods infected with *S. solidus* on day 0, cam: Copepods infected with *C. lacustris* on day 7, SCH-cam: Copepods infected with one *S. solidus* on day 0 plus one *C. lacustris* on day 7.

**Activity after simulated predator attack**														
Day	9		11		13		15		17		19		21	
	Z	p	Z	p	Z	p	Z	p	Z	p	Z	p	Z	p
Control _SCH	-1.17	0.646	-1.79	0.280	0.69	0.900	0.67	0.908	-0.53	0.952	1.10	0.692	0.19	0.998
Control _cam	-4.12	**<0.001**	-7.07	**<0.001**	-8.44	**<0.001**	-8.21	**<0.001**	-0.65	0.914	2.66	**0.039**	1.42	0.489
Control _SCH-cam	-5.68	**<0.001**	-6.93	**<0.001**	-5.09	**<0.001**	-4.00	**<0.001**	-1.70	0.323	1.13	0.672	0.84	0.833
SCH_cam	2.70	0.035	4.85	**<0.001**	8.56	**<0.001**	8.29	**<0.001**	0.07	1	-1.37	0.515	-1.13	0.670
SCH_SCH-cam	-4.21	**<0.001**	-4.85	**<0.001**	-5.39	**<0.001**	-4.35	**<0.001**	-1.04	0.726	-0.05	1	0.59	0.934
cam_SCH-cam	-1.67	0.340	-0.16	0.999	3.78	**0.001**	4.57	**<0.001**	-1.05	0.718	-1.53	0.417	-0.58	0.937
Observations	4470		4320		4500		4500		4410		4470		4410	
Copepods	149		144		150		150		147		149		147	
**Activity after a recovery period**														
Day	9		11		13		15		17		19		21	
	Z	p	Z	p	Z	p	Z	p	Z	p	Z	p	Z	p
Control _SCH	-3.96	**<0.001**	-4.01	**<0.001**	-4.74	**<0.001**	-4.61	**<0.001**	-3.41	**0.004**	-2.48	0.064	-1.30	0.564
Control _cam	-5.3	**<0.001**	-8.16	**<0.001**	-10.3	**<0.001**	-11.56	**<0.001**	-3.23	**0.007**	0.29	0.992	-0.39	0.980
Control _SCH-cam	-7.66	**<0.001**	-8.35	**<0.001**	-9.97	**<0.001**	-11.04	**<0.001**	-5.19	**<0.001**	-1.77	0.288	-1.46	0.459
SCH_cam	1.00	0.749	3.79	**0.001**	5.24	**<0.001**	7.02	**<0.001**	-0.44	0.971	-2.73	**0.032**	-0.92	0.792
SCH_SCH-cam	-3.34	**0.004**	-4.16	**<0.001**	-5.01	**<0.001**	-6.44	**<0.001**	-1.42	0.489	0.85	0.833	-0.06	1
cam_SCH-cam	-2.51	*0.058*	-0.5	0.960	0.18	0.998	0.69	0.899	-2.01	0.184	-2.05	0.170	-1.06	0.715
Observations	4470		4320		4500		4500		4410		4470		4410	
Copepods	149		144		150		150		147		149		147	

Latency to resume moving after a simulated predator attack														
Day	9		11		13		15		17		19		21	
	Z	p	Z	p	Z	p	Z	p	Z	p	Z	p	Z	p
Control _SCH	0.66	0.910	1.15	0.656	-0.53	0.952	-1.04	0.726	-0.17	0.998	-2.16	0.136	-0.11	0.999
Control _cam	2.41	0.075	5.32	**<0.001**	6.48	**<0.001**	7.69	**<0.001**	-0.51	0.957	-4.22	**<0.001**	-0.88	0.815
Control _SCH-cam	5.31	**<0.001**	6.62	**<0.001**	3.41	**0.004**	3.34	**0.005**	-0.79	0.860	-1.89	0.231	-0.57	0.942
SCH_cam	-1.57	0.395	-3.76	**0.001**	-6.53	**<0.001**	-8.15	**<0.001**	0.29	0.991	1.77	0.287	0.71	0.893
SCH_SCH-cam	4.26	**<0.001**	4.98	**<0.001**	3.69	**0.001**	4.13	**<0.001**	-0.55	0.946	0.40	0.978	-0.41	0.976
cam_SCH-cam	2.87	**0.021**	1.37	0.516	-3.07	**0.012**	-4.34	**<0.001**	-0.29	0.992	2.35	0.088	0.32	0.988
Copepods	149		144		150		150		147		149		147	
